# Prediction of functional outcomes of schizophrenia with genetic biomarkers using a bagging ensemble machine learning method with feature selection

**DOI:** 10.1038/s41598-021-89540-6

**Published:** 2021-05-13

**Authors:** Eugene Lin, Chieh-Hsin Lin, Hsien-Yuan Lane

**Affiliations:** 1 Department of Biostatistics, University of Washington, Seattle, WA 98195 USA; 2 Department of Electrical and Computer Engineering, University of Washington, Seattle, WA 98195 USA; 3 Graduate Institute of Biomedical Sciences, China Medical University, Taichung, Taiwan; 4 Department of Psychiatry, Kaohsiung Chang Gung Memorial Hospital, Chang Gung University College of Medicine, Kaohsiung, Taiwan; 5 School of Medicine, Chang Gung University, Taoyüan, Taiwan; 6 Department of Psychiatry, China Medical University Hospital, Taichung, Taiwan; 7 Brain Disease Research Center, China Medical University Hospital, Taichung, Taiwan; 8 Department of Psychology, College of Medical and Health Sciences, Asia University, Taichung, Taiwan

**Keywords:** Computational biology and bioinformatics, Neuroscience, Biomarkers, Molecular medicine

## Abstract

Genetic variants such as single nucleotide polymorphisms (SNPs) have been suggested as potential molecular biomarkers to predict the functional outcome of psychiatric disorders. To assess the schizophrenia’ functional outcomes such as Quality of Life Scale (QLS) and the Global Assessment of Functioning (GAF), we leveraged a bagging ensemble machine learning method with a feature selection algorithm resulting from the analysis of 11 SNPs (*AKT1* rs1130233, *COMT* rs4680, *DISC1* rs821616, *DRD3* rs6280, *G72* rs1421292, *G72* rs2391191, *5-HT2A* rs6311, *MET* rs2237717, *MET* rs41735, *MET* rs42336, and *TPH2* rs4570625) of 302 schizophrenia patients in the Taiwanese population. We compared our bagging ensemble machine learning algorithm with other state-of-the-art models such as linear regression, support vector machine, multilayer feedforward neural networks, and random forests. The analysis reported that the bagging ensemble algorithm with feature selection outperformed other predictive algorithms to forecast the QLS functional outcome of schizophrenia by using the *G72* rs2391191 and *MET* rs2237717 SNPs. Furthermore, the bagging ensemble algorithm with feature selection surpassed other predictive algorithms to forecast the GAF functional outcome of schizophrenia by using the *AKT1* rs1130233 SNP. The study suggests that the bagging ensemble machine learning algorithm with feature selection might present an applicable approach to provide software tools for forecasting the functional outcomes of schizophrenia using molecular biomarkers.

## Introduction

Precision psychiatry is a newly-developed interdisciplinary study of precision medicine and psychiatry^1,2^, where state-of-the-art artificial intelligence and machine learning methods are integrated with molecular biomarkers such as genetic variants to provide personalized arrangements during all phases of medical intervention^3–6^. For instance, studies in precision psychiatry using machine learning algorithms involve the prediction of diagnosis of schizophrenia^7,8^ and the prediction of treatment response in patients with major depressive disorder^9,10^. On another note, functional outcomes in schizophrenia, which are normally determined by the tools such as Quality of Life Scale (QLS)^11^ and the Global Assessment of Functioning (GAF) Scale^12^, may affect the diagnosis and treatment of schizophrenia patients. As a result, it is vital to establish potential biomarkers that affect functional outcomes in schizophrenia^13^. Accordingly, we hypothesized that machine learning algorithms may be capable of forecasting probable biomarkers that influence functional outcomes in schizophrenia by using molecular biomarkers such as genetic variants.

Genetic variants such as single nucleotide polymorphisms (SNPs) have been a focus of attention in schizophrenia research. Various SNPs have been indicated as potential molecular biomarkers with respect to the developmental etiology of schizophrenia (Supplementary Table S1), including *AKT1* rs1130233, *COMT* rs4680, *DISC1* rs821616, *DRD3* rs6280, *G72* rs1421292, *G72* rs2391191, *5-HT2A* rs6311, *MET* rs2237717, *MET* rs41735, *MET* rs42336, and *TPH2* rs4570625. For example, a previous association study by Emamian et al.^14^ indicated that there was a significant association of schizophrenia with the rs1130233 variant in the *AKT1* gene. Another study by Chen et al.^15^ also reported that *COMT* rs4680 contributed to schizophrenia in Irish patents. In addition, a study by Callicott et al.^16^ showed that *DISC1* rs821616 significantly influenced hippocampal structure and increased the risk for schizophrenia. Moreover, Talkowski et al.^17^ implicated that *DRD3* rs6280 was markedly associated with schizophrenia in the U.S. samples. In order to differentiate schizophrenia patients from healthy individuals, Lin et al.^8^ employed machine learning algorithms (such as logistic regression, naive Bayes, and C4.5 decision tree) to construct classification models by using *G72* rs1421292, *G72* rs2391191, and G72 protein. Furthermore, a link between *5-HT2A* rs6311 and a sensorimotor gating deficit in schizophrenia was observed in schizophrenia patients^18^. Additionally, Burdick et al.^19^ detected the association of *MET* rs2237717, *MET* rs41735, and *MET* rs42336 with schizophrenia risk and general cognitive ability in schizophrenia patients. The association of *TPH2* rs4570625 with schizophrenia was not statistically significant in Korean schizophrenia patients^20^; however, it was related with social cognition^21^.

In a previous study, Lin et al.^13^ reported that clinical symptoms contribute to the link between cognitive behaviors and functional outcomes in schizophrenia by applying the structural equation modeling method. Additionally, it has been suggested that machine learning methods incorporating with feature selection techniques possess the advantages of improved prediction in precision psychiatry studies^10,22,23^. Here, we employed the same cohort of 302 schizophrenia patients and performed the first study on the QLS and GAF functional outcome prediction in schizophrenia with 11 aforementioned molecular biomarkers (namely 11 SNPs) by using a bagging ensemble machine learning method^24^. Moreover, in order to predict functional outcomes with improved performance, we utilized the M5 Prime feature selection algorithm^25^ to identify a small subset of suitable biomarkers from the 11 SNPs. We inferred that our bagging ensemble machine learning method would be capable of forecasting the QLS and GAF functional outcomes of schizophrenia by utilizing a small subset of chosen genetic variants. To the best of our knowledge, no preceding studies have been conducted to assess predictive algorithms for functional outcomes in schizophrenia with molecular biomarkers by utilizing the bagging ensemble machine learning method with the M5 Prime feature selection algorithm. We chose the bagging ensemble machine learning method due to its merits in lower variance and less overfitting; and thereby this method is widely leveraged to deal with complicated prediction and classification studies^24,25^. This study precisely scrutinized the performance of the bagging ensemble machine learning method to other broadly-used machine learning models, including support vector machine (SVM), multi-layer feedforward neural networks (MFNNs), linear regression, and random forests. The analysis showed that the bagging ensemble machine learning method with the M5 Prime feature selection algorithm led to improved performance.

## Results

### The functional outcomes of the study cohort

The participants encompassed 302 schizophrenia patients in the Taiwanese population. Study measures in regard to demographic characteristics and the QLS and GAF of schizophrenia were detailed before^13^.

### Genetic variants

There were 11 genetic variants including *AKT1* rs1130233, *COMT* rs4680, *DISC1* rs821616, *DRD3* rs6280, *G72* rs1421292, *G72* rs2391191, *5-HT2A* rs6311, *MET* rs2237717, *MET* rs41735, *MET* rs42336, and *TPH2* rs4570625. Their genotype frequencies are shown in Table 1. All of them, except *G72* rs1421292, did not deviate from the Hardy–Weinberg equilibrium.

**Table 1:** Genotype frequencies of 11 genetic polymorphisms in 302 schizophrenia patients.

Genetic polymorphisms	Genotype frequency	*P* value of Hardy–Weinberg equilibrium
*AKT1* rs1130233	AA/AG/GG: 0.31/0.49/0.20	0.899
*COMT* rs4680	GG/GA/AA: 0.56/0.35/0.09	0.066
*DISC1* rs821616	TT/TA/AA: 0.79/0.20/0.01	0.676
*DRD3* rs6280	AA/AG/GG: 0.48/0.45/0.07	0.170
*G72* rs1421292	TT/TA/AA: 0.41/0.42/0.18	0.040
*G72* rs2391191	AA/AG/GG: 0.37/0.49/0.14	0.597
*5-HT2A* rs6311	AA/AG/GG: 0.36/0.51/0.13	0.133
*MET* rs2237717	CC/CT/TT: 0.30/0.46/0.24	0.183
*MET* rs41735	GG/GA/AA: 0.31/0.48/0.21	0.593
*MET* rs42336	AA/GA/GG: 0.30/0.48/0.22	0.578
*TPH2* rs4570625	TT/GT/GG: 0.25/0.51/0.24	0.814

### Feature selection using genetic variants

We completed a series of various biomarker combinations using the 11 genetic variants (Table 2; the Feature-A–Feature-C sets) to forecast the QLS and GAF of schizophrenia. Note that the Feature-A set encompasses the 11 genetic variants.

**Table 2:** The results of repeated tenfold cross-validation experiments for predicting the QLS and GAF functional outcome of schizophrenia with genetic variants using machine learning predictors such as the bagging ensemble model with feature selection, the bagging ensemble model, MFNNs, SVM, linear regression, and random forests.

Algorithm	QLS			GAF		
	RMSE	Feature set	Number of features	RMSE	Feature set	Number of features
Bagging ensemble with feature selection	**8.6766 ± 1.0421**	Feature-B	2	**9.6982 ± 1.3354**	Feature-C	1
Bagging ensemble	8.7102 ± 1.0716	Feature-A	11	9.7777 ± 1.3301	Feature-A	11
SVM	8.8799 ± 1.0893	Feature-A	11	10.0754 ± 1.4486	Feature-A	11
MFNNs	8.8675 ± 1.1103	Feature-A	11	10.0625 ± 1.3753	Feature-A	11
Linear regression	8.7839 ± 1.0538	Feature-A	11	9.7011 ± 1.3341	Feature-A	11
Random forests	9.4253 ± 1.1750	Feature-A	11	10.4998 ± 1.3586	Feature-A	11

The best QLS or GAF score is shown in bold.

Feature-A: 11 features (related to 11 SNPs) including *AKT1* rs1130233, *COMT* rs4680, *DISC1* rs821616, *DRD3* rs6280, *G72* rs1421292, *G72* rs2391191, *5-HT2A* rs6311, *MET* rs2237717, *MET* rs41735, *MET* rs42336, and *TPH2* rs4570625.

Feature-B: 2 features (related to 2 SNPs) including *G72* rs2391191 and *MET* rs2237717.

Feature-C: 1 feature (related to 1 SNP) including *AKT1* rs1130233.

*GAF* Global assessment of functioning, *MFNNs* Multilayer feedforward neural networks, *QLS* Quality of life scale, *RMSE* Root mean square error, *SNPs* Single nucleotide polymorphisms, *SVM* Support vector machine.

Data are presented as mean ± standard deviation.

First, for forecasting the QLS, we utilized the M5 Prime feature selection algorithm (see Methods) to find two biomarkers (such as *G72* rs2391191 and *MET* rs2237717) from the 11 genetic variants, where the Feature-B dataset comprises these two selected biomarkers (Supplementary Figure S1).

Second, for forecasting the GAF, we utilized the M5 Prime feature selection algorithm to identify one biomarker (such as *AKT1* rs1130233) from the 11 genetic variants, where the Feature-C dataset comprises this selected biomarker (Supplementary Figure S2).

### Prediction of the QLS and GAF of schizophrenia using genetic variants

We utilized genetic variants (namely the Feature-A–Feature-C datasets) to create the predictive algorithms for the QLS and GAF of schizophrenia, respectively. Table 2 shows the results of repeated tenfold cross-validation experiments for the predictive algorithms using genetic variants by the bagging ensemble algorithm with feature selection (Supplementary Figures S1 and S2), the bagging ensemble algorithm (Supplementary Figure S3), SVM (Supplementary Figure S4), MFNNs (Supplementary Figure S5), linear regression (Supplementary Figure S6), and random forests (Supplementary Figure S7). Furthermore, we utilized the RMSE values to assess the performance of the predictive algorithms.

As shown in Table 2, to forecast the QLS, the bagging ensemble algorithm with feature selection (Supplementary Figure S1) obtained the RMSE value of 8.6766 ± 1.0421 using the Feature-B dataset (namely *G72* rs2391191 and *MET* rs2237717).

Moreover, to forecast the GAF, the bagging ensemble algorithm with feature selection (Supplementary Figure S2) obtained the RMSE value of 9.6982 ± 1.3354 using the Feature-C dataset (namely *AKT1* rs1130233) (Table 2).

### Benchmarking

We scrutinized the results (Table 2) for forecasting the QLS of schizophrenia among machine learning predictive models including the bagging ensemble algorithm with feature selection (Supplementary Figure S1), the bagging ensemble algorithm (Supplementary Figure S3), SVM (Supplementary Figure S4), MFNNs (Supplementary Figure S5), linear regression (Supplementary Figure S6), and random forests (Supplementary Figure S7) using two biomarker datasets (namely Feature-A and Feature-B). We found that the bagging ensemble algorithm with feature selection (using Feature-B; Supplementary Figure S1) performed best to forecast the QLS. The best RMSE value for forecasting the QLS was 8.6766 ± 1.0421 (Table 2).

In addition, we scrutinized the results (Table 2) for forecasting the GAF of schizophrenia among machine learning predictive models including the bagging ensemble algorithm with feature selection (Supplementary Figure S2), the bagging ensemble algorithm (Supplementary Figure S3), SVM (Supplementary Figure S4), MFNNs (Supplementary Figure S5), linear regression (Supplementary Figure S6), and random forests (Supplementary Figure S7) using two biomarker datasets (namely Feature-A and Feature-C). We found that the bagging ensemble algorithm with feature selection (using Feature-C; Supplementary Figure S2) performed best to forecast the GAF. The best RMSE value for forecasting the GAF was 9.6982 ± 1.3354 (Table 2).

Here, we observed that the bagging ensemble algorithm with feature selection using the chosen biomarkers from SNPs achieved best outcome forecasting in terms of both QLS and GAF when compared to other state-of-the-art models, including SVM, MFNNs, linear regression, and random forests. Our analysis suggested that the bagging ensemble algorithm with feature selection was well-adapted for predictive algorithms in the functional outcomes of schizophrenia.

## Discussion

To our knowledge, this is the first study to date to explore a bagging ensemble machine learning method with the M5 Prime feature selection algorithm using molecular biomarkers for constructing predictive algorithms of functional outcomes in schizophrenia among Taiwanese patients. In addition, we conducted the first study to search probable biomarkers for functional outcomes of schizophrenia by using genetic biomarkers. The findings indicated that the bagging ensemble machine learning method with feature selection using two genetic biomarkers (*G72* rs2391191 and *MET* rs2237717 SNPs) surpassed other state-of-the-art predictive models in terms of RMSE for forecasting the QLS outcome. Moreover, for forecasting the GAF outcome, we observed that the bagging ensemble machine learning method with feature selection using one genetic biomarker (*AKT1* rs1130233) surpassed other state-of-the-art predictive algorithms in terms of RMSE.

By taking advantage of the genetic biomarkers, we created the predictive algorithms of functional outcomes in schizophrenia patients using the bagging ensemble machine learning method with the M5 Prime feature selection algorithm. This study is a proof of concept of a machine learning predictive framework for forecasting functional outcomes of schizophrenia. The results suggest that the bagging ensemble machine learning method may provide a clinically feasible tool for predicting functional outcomes of schizophrenia.

In addition, it is worthwhile to discuss the M5 Prime feature selection algorithm for discovering probable biomarkers in this study. We found that the bagging ensemble machine learning method with the selected biomarkers of the M5 Prime feature selection algorithm consistently surpassed the bagging ensemble machine learning method without using feature selection. For example, the bagging ensemble machine learning method with the Feature-B dataset excelled the bagging ensemble machine learning method with the Feature-A in forecasting the QLS outcome. Likewise, the bagging ensemble model with the Feature-C dataset surpassed the bagging ensemble machine learning method with the Feature-A dataset in forecasting the GAF outcome. In other words, the bagging ensemble machine learning method with feature selection inclined to obtain lower RMSE values (the better the performance). The findings suggest that the M5 Prime feature selection algorithm may have a better potential to single out biomarkers affecting functional outcomes of schizophrenia. In accordance, it has been reported that machine learning methods with feature selection outperformed the ones without feature selection in predicting the diagnosis and treatment outcome of psychiatric disorders^10,22,23^.

Remarkably, we further speculated the synergistic effects of chosen biomarkers (namely the Feature-B dataset), which were pinpointed by the M5 Prime feature selection algorithm when a biomarker dataset of 11 genetic variants was utilized to forecast the QLS outcome. As indicated in “Results” section the Feature-B dataset comprised 2 SNPs (namely *G72* rs2391191 and *MET* rs2237717) for the QLS outcome. Subsequently, the bagging ensemble machine learning method with feature selection using the Feature-B dataset performed best in predicting the QLS outcome among the predictive algorithms. To our knowledge, scanty studies have been investigated to assess causal links between genetic variants. The biological mechanisms of these causal links in the functional outcomes of schizophrenia remain to be elucidated. It has been demonstrated that *MET* rs2237717 was linked to schizophrenia^19^ and *G72* rs2391191 was also associated with schizophrenia^8^. Based on the previous findings^8,19^, it is hypothesized that synergistic interactions between genetic variants may provide a hallmark of molecular effects on the functional outcomes of schizophrenia.

In conclusion, we built a bagging ensemble machine learning method with feature selection for predicting functional outcomes of schizophrenia in Taiwanese patients by using genetic biomarkers. The analysis reveals that the bagging ensemble machine learning method with feature selection may present a plausible tool to construct predictive models for functional outcomes of schizophrenia in terms of favorable performance. Nonetheless, it is fundamental to further investigate the role of the bagging ensemble machine learning method by more replication studies. Ultimately, we would expect that the findings of the present study may be generalized in precision psychiatry to predict the diagnosis and treatment outcomes for various psychiatric disorders. Furthermore, the findings may be presumably leveraged to develop molecular diagnostic and prognostic tools in the near future.

## Materials and methods

### Study population

The study cohort composed of 302 schizophrenia patients, who were recruited from the China Medical University Hospital and affiliated Taichung Chin-Ho Hospital in Taiwan^13^. In this study, schizophrenia patients were aged 18–65 years and were healthy in the physical conditions. After presenting a complete description of this study to the subjects, we obtained written informed consents from a parent and/or legal guardian in line with the institutional review board guidelines. Details of the diagnosis of schizophrenia were published previously^13^. This study was approved by the institutional review board of the China Medical University Hospital in Taiwan and was performed in accordance with the Declaration of Helsinki.

### Functional outcomes

We assessed functional outcomes by employing the QLS^11^ and the GAF Scale of the DSM-IV^12^. The QLS is a clinical tool for assessing the functional outcomes in patients with schizophrenia, including anhedonia, aimless inactivity, capacity for empathy, curiosity, emotional interaction, motivation, sense of purpose, social activity, social initiatives, and social withdrawal^11^. The GAF is a clinical tool for evaluating the global psychological, social, and occupational functioning in patients with schizophrenia^12^.

### Laboratory assessments: genotyping

DNA was extracted from venous blood. In this study, the panel of genetic variants consisted of the aforementioned 11 SNPs. Their genotyping methods were detailed previously: *AKT1* rs1130233^26^, *COMT* rs4680^21^, *DISC1* rs821616^27^, *DRD3* rs6280^28^, *G72* rs1421292^8^, *G72* rs2391191^8^, *5-HT2A* rs6311^29^, *MET* rs2237717^26^, *MET* rs41735^26^, *MET* rs42336^26^, and *TPH2* rs4570625^21^. These 11 genetic variants were used to create the predictive algorithms for the QLS and GAF of schizophrenia.

### Statistical analysis

For genetic variants, we assessed the genotype frequencies for Hardy–Weinberg equilibrium by using a chi-squared goodness-of-fit test with 1 degree of freedom^30^. The criterion for failure to achieve Hardy–Weinberg equilibrium was set at *P* < 0.05. Data are presented as the mean ± standard deviation.

### Bagging ensemble machine learning method

We applied a key ensemble machine learning method called bagging predictors^24^ and employed the Waikato Environment for Knowledge Analysis (WEKA) software (which is available from https://www.cs.waikato.ac.nz/ml/weka/)25 to conduct the bagging ensemble machine learning method. All the experiments were carried out on a computer with Intel (R) Core (TM) i5-4210U, 4 GB RAM, and Windows 7^7^.

In principle, the bagging ensemble machine learning method (Supplementary Figure S3) takes advantage of averaging the predictive performance of multiple versions of a base model to obtain a combined model with better performance^24^. The multiple versions of the base model are generated by bootstrap reproductions, where the bootstrap technique is one of the most suitable data resampling approaches employed in statistical analysis. In other words, the bootstrap technique produces the multiple versions of the base model, that is, the Model-version #1 to the Model-version #n (Supplementary Figure S3). Subsequently, the combined model summarizes the predictive performance of these base models from 1 to n. The technique of bagging models inclines to lower variance and prevent overfitting. The base model we used was linear regression. Here, we utilized the default tuning parameters of WEKA, such as 100 for the batch size, 100 for the percentage of the bag size, and 10 for the number of iterations^7,10^.

**Figure 1:**
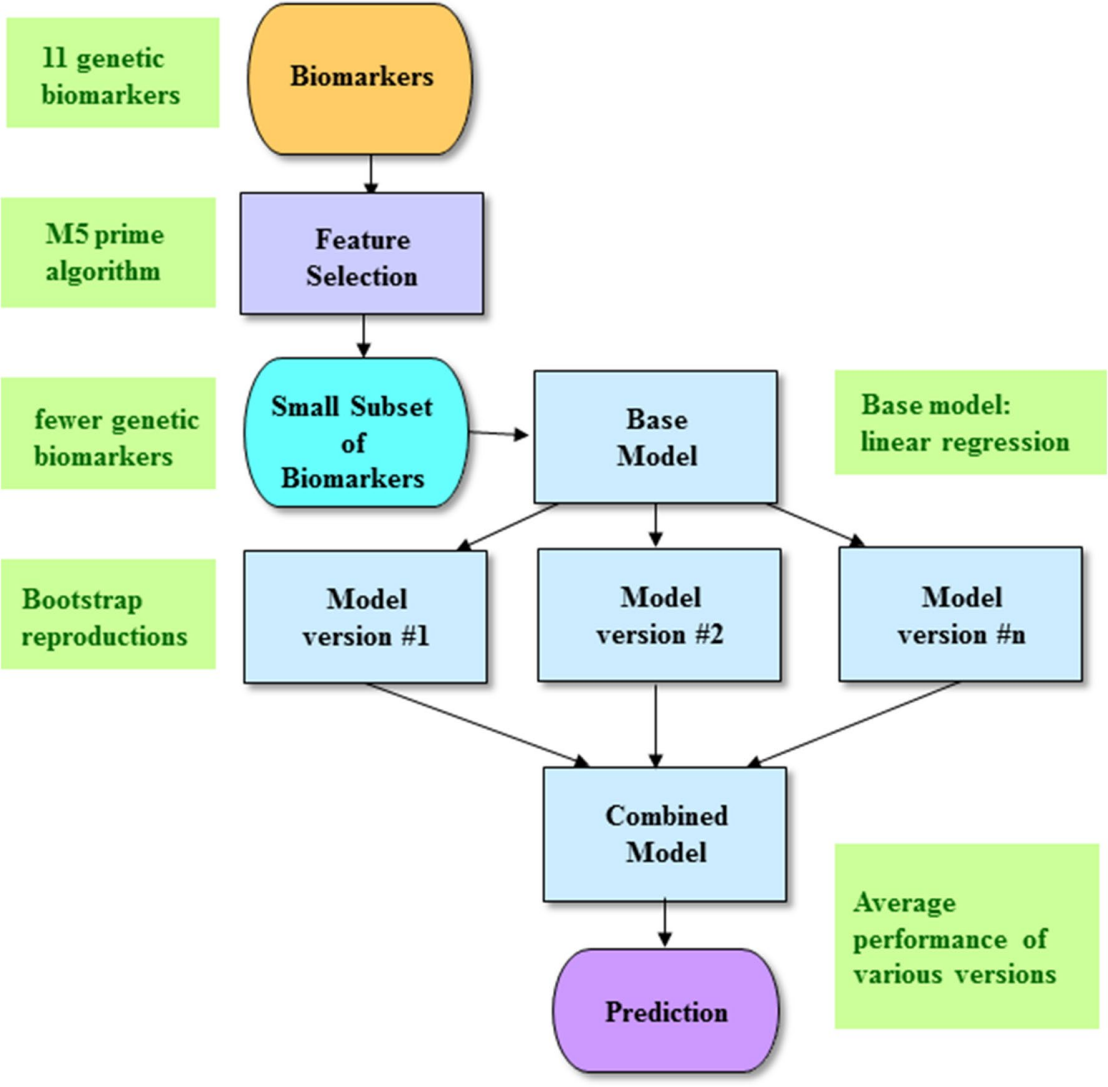
The schematic illustration of the bagging ensemble machine learning method with feature selection. First, the M5 Prime feature selection algorithm is conducted to find a small subset of biomarkers, which serves as the input to the bagging ensemble machine learning method. The concept of the bagging ensemble machine learning method is to create the multiple versions of a base model by bootstrap reproductions. Then, the ultimate prediction is generated by averaging the predictive performance of the multiple versions. The base model was chosen as linear regression in this study.

Figure 1 demonstrates the illustrative diagram of the bagging ensemble machine learning method with feature selection. For the feature selection task, we utilized the M5 Prime algorithm (as described below).

### M5 Prime feature selection algorithm

In the present study, we used an Akaike information criterion (AIC)-based method called the M5 Prime algorithm^25,31^ for the feature selection function. The M5 Prime algorithm builds a decision tree with multivariate linear models at the terminal nodes and iteratively eliminates the biomarker with the smallest normalized coefficient until no further improvement in the evaluated error specified by the AIC^32,33^. We chose the M5 Prime algorithm due to its merits in dealing with the large number of biomarkers, performing fast during training, and being a straightforward approach^25,31^. In addition, the relevant features of the M5 Prime algorithm include robustness in handling missing values and enumerated attributes^25,31^.

To forecast the QLS and GAF, we utilized the M5 Prime algorithm to choose biomarkers from a biomarker dataset, which includes 11 genetic variants (Fig. 1). By using 11 genetic variants, the M5 Prime algorithm generated the first feature dataset including two genetic variants (Supplementary Figure S1). In addition, by using 11 genetic variants, the M5 Prime algorithm generated the second feature dataset including one genetic variant (Supplementary Figure S2).

### Machine learning algorithms for benchmarking

For the benchmarking task in the present study, we employed four state-of-the-art machine learning models including SVM, MFNNs, linear regression, and random forests (Supplementary Figures S4–S7). We performed the analyses for these four machine learning models using the WEKA software^25^ and a computer with Intel (R) Core (TM) i5-4210U, 4 GB RAM, and Windows 7^7^.

First, the SVM model^34^ (Supplementary Figure S4) is a popular approach for pattern recognition and classification^7,35–37^. Given a training set, the SVM model applies a kernel function to find a linear relationship between input variables and the predicted output^34,38^. The SVM model then determines the best predicted output by minimizing both the coefficients of the cost function and the predictive errors^34,38^. In this study, we utilized the WEKA’s tuning parameter for the polynomial kernel with the exponent value of 1.0^7,10^.

Second, an MFNN model (Supplementary Figure S5) comprises one input layer, one or multiple hidden layers, and one output layer, where links among neuron nodes actually have no directed cycles^7,39^. In the learning stage of the MFNN model, the back-propagation algorithm^40^ is achieved for the learning task. In the retrieval stage, the MFNN model reiterates by way of all the neuron nodes to accomplish the retrieval task at the output layer based on the inputs of test data^7,41^. In this study, we utilized the architecture incorporating one hidden layer. For instance, we utilized the following WEKA’s tuning parameters for training the MFNN model with one hidden layer: the momentum = 0.01, the learning rate = 0.01, and the batch size = 100^7,42^.

Next, the linear regression model (Supplementary Figure S6), the conventional approach for prediction issues in clinical studies, was utilized as a basis for the benchmarking task^7,25^.

Finally, the random forests model (Supplementary Figure S7) is an ensemble learning approach which consists of a group of decision trees throughout training and produces a better prediction by aggregating the predictive results among the individual decision trees^7,35–37,43^. Here, we utilized the default tuning parameters of WEKA for the random forests model; for instance, 100 for the batch size and 100 for the number of iterations^7^.

### Evaluation of the predictive performance

In this study, we employed one of the most popular standards, the root mean square error (RMSE), to examine the performance of predictive algorithms^22,38,44^. The RMSE estimates the difference between the measured values and the predicted values by a predictive algorithm. The better the prediction algorithm, the lower the RMSE^22,44^. In addition, we applied the repeated tenfold cross-validation method to assess the generalization of predictive models^45^. Firstly, the whole dataset was randomly fragmented into ten individual partitions. Secondly, the predictive model was trained using nine-tenths of the partitions and was tested using the remaining tenth of the partitions to estimate the predictive performance. Next, the previous step was repeated nine more times by choosing different nine-tenths of the partitions for training and a different tenth of the partitions for testing. Lastly, the final estimation was evaluated by averaging the aforementioned ten runs. In the present study, we reported the performance of all predictive models using the repeated tenfold cross-validation method.

## Supplementary Information

Supplementary Information 1.

Supplementary Information 2.

## Data Availability

All data generated or analyzed during this study are included in this published article.
