# Supplementary material for: Prediction of functional outcomes of schizophrenia with genetic biomarkers using a bagging ensemble machine learning method with feature selection
Source: Sci Rep. 2021 May 13;11:10179. doi: 10.1038/s41598-021-89540-6 (PMC8119477; doi:10.1038/s41598-021-89540-6)
Supplement: Supplementary file 1 — Supplementary Information 1. [file 41598_2021_89540_MOESM1_ESM.pdf]

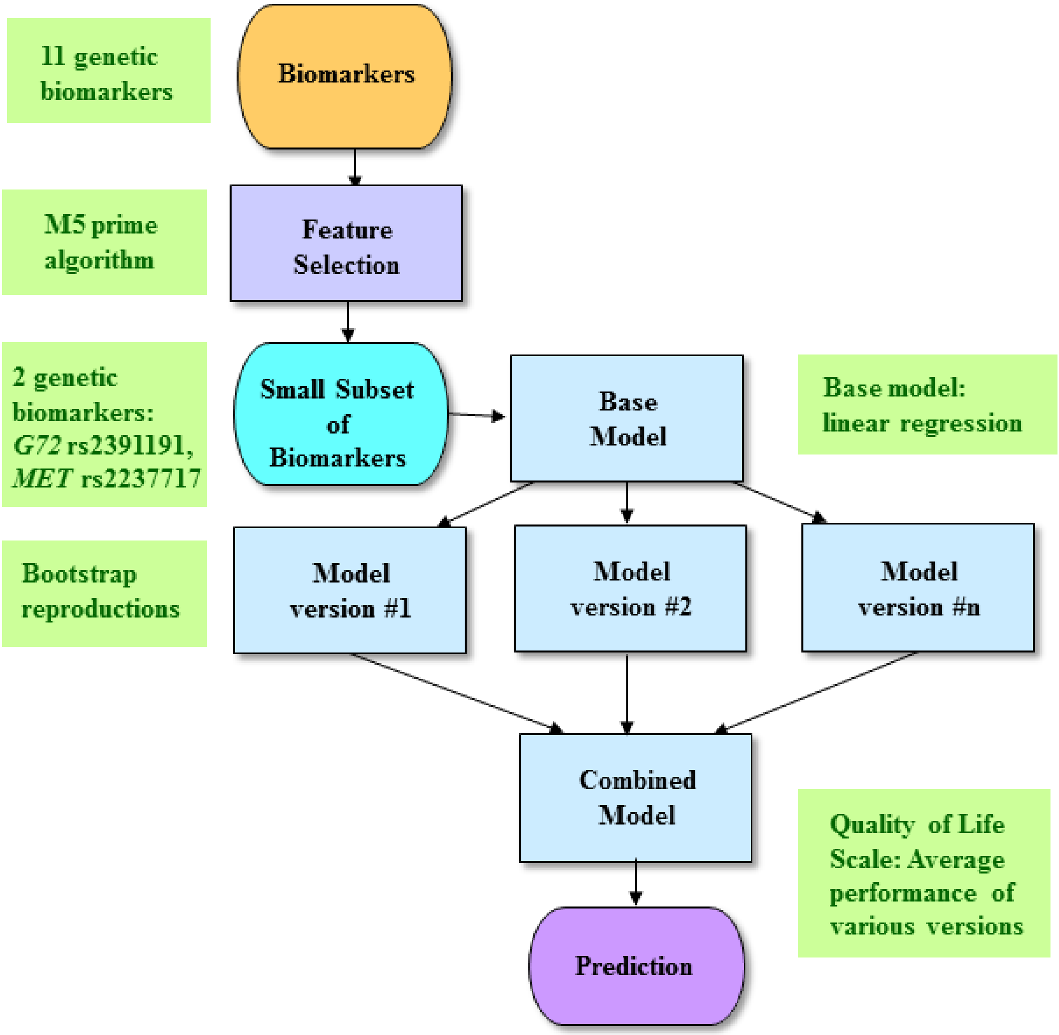

Figure S1. The schematic illustration of the bagging ensemble machine learning method with feature selection for forecasting the Quality of Life Scale (QLS). First, the M5 Prime feature selection algorithm is conducted to find two biomarkers (such as *G72* rs2391191 and *MET* rs2237717) from the 11 genetic variants. These two biomarkers serve as the input to the bagging ensemble machine learning method. Next, the bagging ensemble machine

learning method creates the multiple versions of a base model (that is, linear regression in this study). Then, the ultimate prediction of the QLS is generated by averaging the predictive performance of the multiple versions.

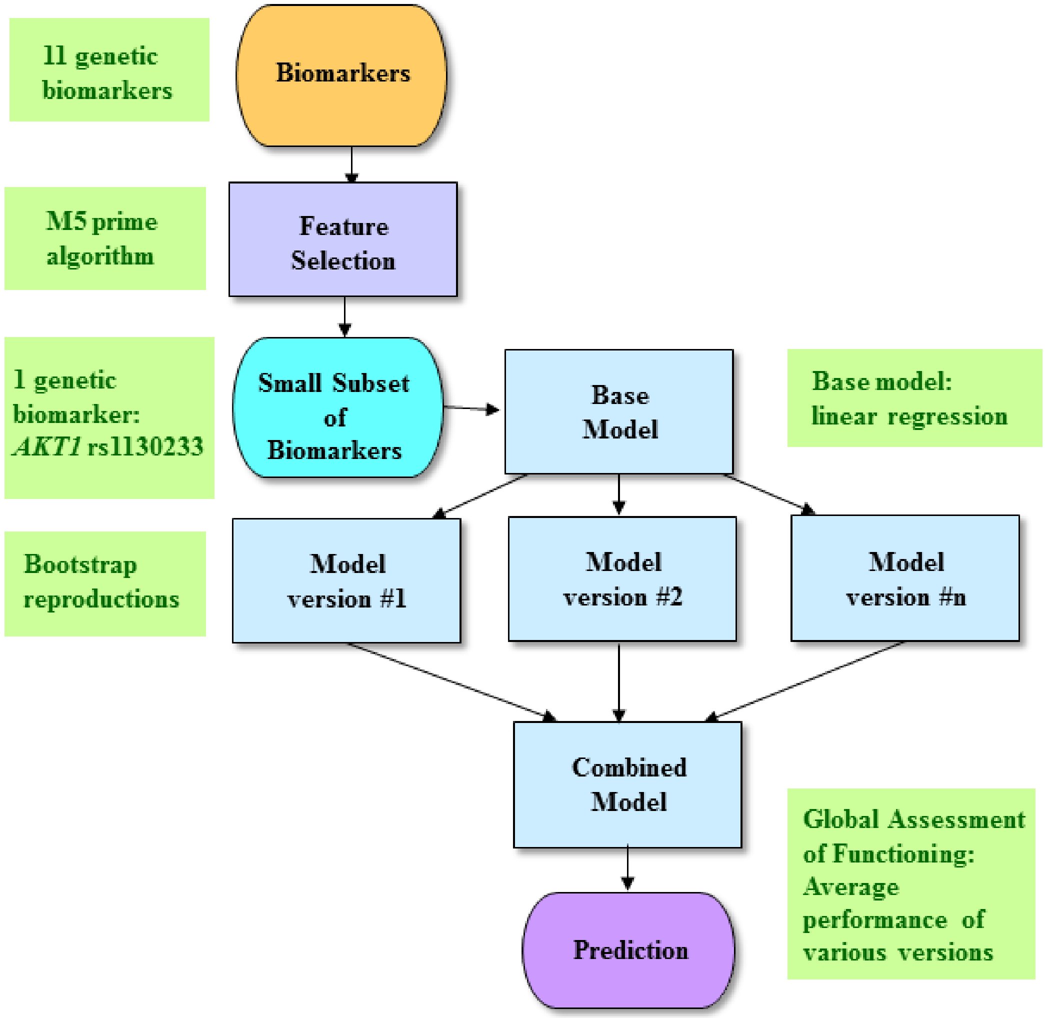

Figure S2. The schematic illustration of the bagging ensemble machine learning method with feature selection for forecasting the Global Assessment of Functioning (GAF). First, the M5 Prime feature selection algorithm is conducted to find one biomarker (such as *AKT1* rs1130233) from the 11 genetic variants. This biomarker serves as the input to the bagging ensemble machine learning method. Next, the bagging ensemble machine learning method creates the multiple versions of a base model (that is, linear regression in this study). Then,

the ultimate prediction of the GAF is generated by averaging the predictive performance of the multiple versions.

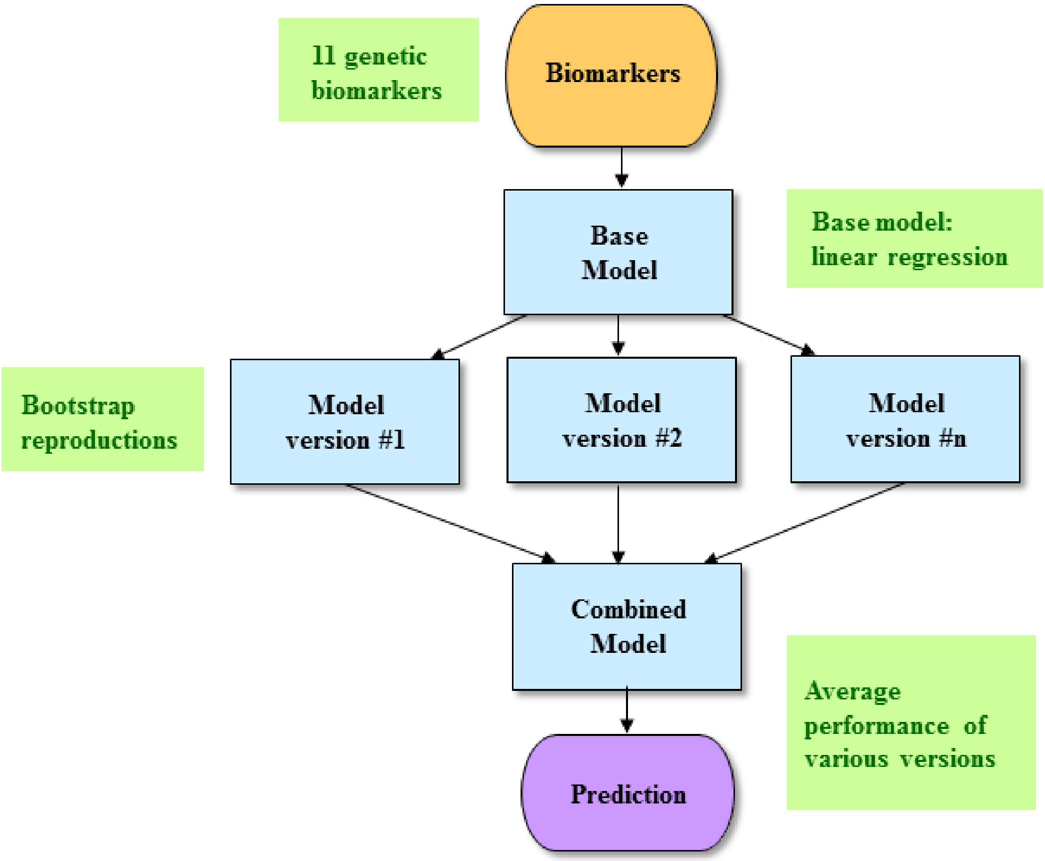

Figure S3. The schematic illustration of the bagging ensemble machine learning method for forecasting the Quality of Life Scale (QLS) or the Global Assessment of Functioning (GAF). The concept of the bagging ensemble machine learning method is to create the multiple versions of a base model by bootstrap reproductions. Then, the ultimate prediction is generated by averaging the predictive performance of the multiple versions. The base model was chosen as linear regression in this study.

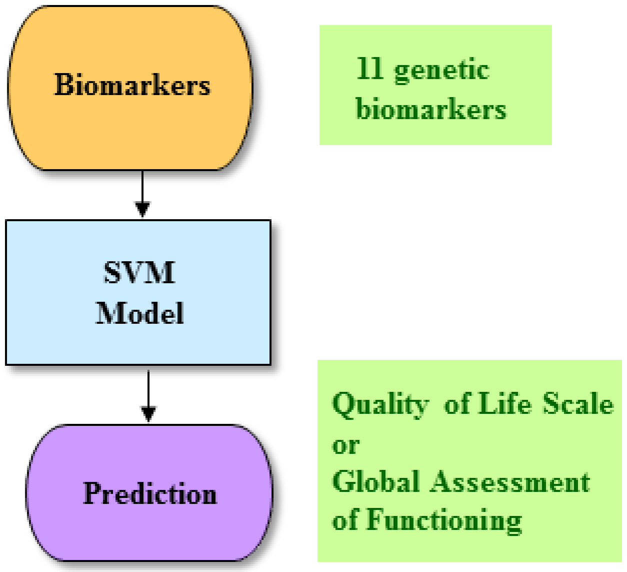

Figure S4. The schematic illustration of the support vector machine (SVM) model for forecasting the Quality of Life Scale (QLS) or the Global Assessment of Functioning (GAF).

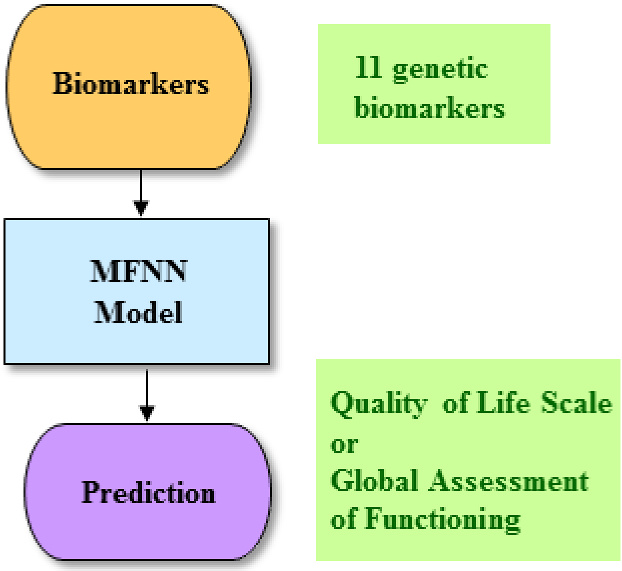

Figure S5. The schematic illustration of the multi-layer feedforward neural network (MFNN) model for forecasting the Quality of Life Scale (QLS) or the Global Assessment of Functioning (GAF).

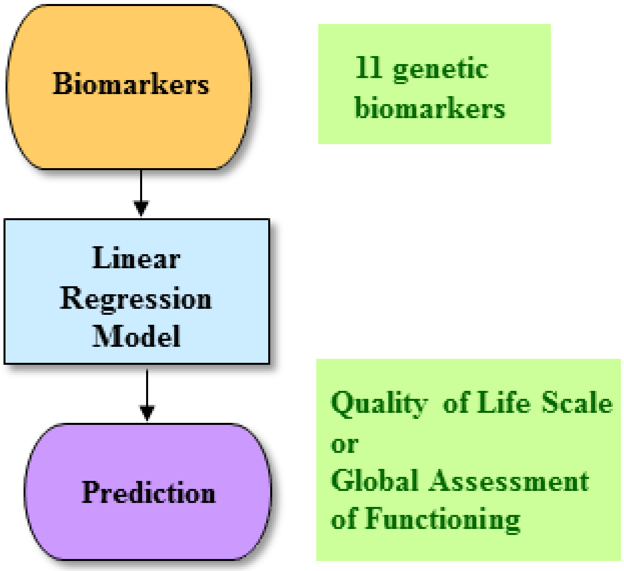

Figure S6. The schematic illustration of the linear regression model for forecasting the Quality of Life Scale (QLS) or the Global Assessment of Functioning (GAF).

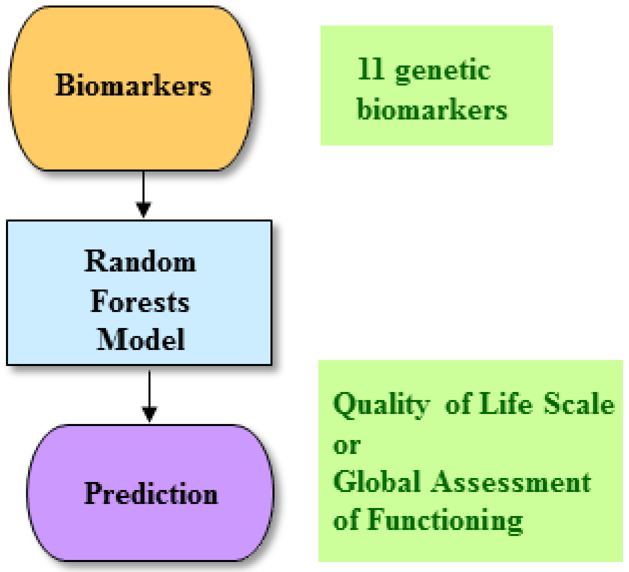

Figure S7. The schematic illustration of the random forests model for forecasting the Quality of Life Scale (QLS) or the Global Assessment of Functioning (GAF).
