# Supplementary material for: Prediction of functional outcomes of schizophrenia with genetic biomarkers using a bagging ensemble machine learning method with feature selection
Source: Sci Rep. 2021 May 13;11:10179. doi: 10.1038/s41598-021-89540-6 (PMC8119477; doi:10.1038/s41598-021-89540-6)
Supplement: Supplementary file 2 — Supplementary Information 2. [file 41598_2021_89540_MOESM2_ESM.pdf]

Table S1. The related studies of using various SNPs as potential molecular biomarkers with respect to the developmental etiology of schizophrenia.

SNPs	Study	Findings
AKT1 rs1130233	Emamian et al. [14]	A significant association with schizophrenia
COMT rs4680	Chen et al. [15]	A significant association with schizophrenia in Irish patents
DISC1 rs821616	Callicott et al. [16]	A significant association with hippocampal structure and schizophrenia
DRD3 rs6280	Talkowski et al. [17]	A significant association with schizophrenia in the U.S. samples
G72 rs1421292,<br>*G72* rs2391191, and<br>G72 protein	Lin et al. [8]	To differentiate schizophrenia patients from healthy individuals using logistic regression, naive Bayes, and C4.5 decision tree.
5-HT2A rs6311	Quednow et al. [18]	A link with a sensorimotor gating deficit in schizophrenia
MET rs2237717,<br>*MET* rs41735, and<br>*MET* rs42336	Burdick et al. [19]	A significant association with schizophrenia risk and general cognitive ability in schizophrenia patients
TPH2 rs4570625	Serretti et al. [20]	No association with schizophrenia in Korean patients
TPH2 rs4570625	Lin et al. [21]	A significant association with social cognition in healthy adults

SNPs = single nucleotide polymorphisms.
